# Task demands and visual context naturalness modulate gravitational expectation during ocular tracking of temporarily occluded ballistic trajectories

**DOI:** 10.1371/journal.pone.0328432

**Published:** 2026-06-03

**Authors:** Sergio Delle Monache, Pietro Nastasi, Gianluca Paolocci, Francesco Scalici, Riccardo Ingrosso, Francesco Lacquaniti, Iole Indovina, Gianfranco Bosco

**Affiliations:** 1 Laboratory of Neuromotor Physiology, IRCCS Santa Lucia Foundation, Rome, Italy; 2 Department of Systems Medicine and Centre of Space Bio-medicine, University of Rome Tor Vergata, Rome, Italy; 3 Department of Medical, Surgical and Technological Sciences, University of Catania, Catania, Italy; University of Giessen: Justus-Liebig-Universitat Giessen, GERMANY

## Abstract

When intercepting a moving object, ocular and hand movements are often associated. The neural control of both tasks is known to combine sensory information with predictive mechanisms. When interacting with free-falling targets, the brain may integrate visual signals with an internal representation of gravity, which can be recruited also in virtual settings, when contextual cues about the environment naturalness are available. This internal representation may be shared between oculomotor and manual control. At the same time, concurrent execution of hand and eye movements may alter the oculomotor performance. Here, we investigated how task demands and visual context naturalness affected the relative weighting of sensory feedback and predictive information for ocular tracking control. Participants tracked targets, either congruent with gravity or perturbed with altered gravity, which were projected on either pictorial or neutral background. To enforce prediction, targets were occluded for variable intervals. One group of participants performed only ocular tracking, while another group also intercepted the targets. In general, subjects’ ocular tracking depended on the target acceleration and the visual context. The effects of gravitational expectations were more evident for the time dependent oculomotor indexes (smooth pursuit and saccadic τ) and with the pictorial background, reinforcing the idea that recruitment of the gravity prior depends on the visual context. Instead, for post saccadic error and smooth pursuit gain no clear dissociation emerged between the effects of acceleration and velocity. Finally, participants who only tracked the target showed strong reliance on gravitational expectation early on, by displaying systematic oculomotor changes with the target acceleration since its perturbation. Instead, participants that also intercepted the target relied less on the gravity prior until the target occlusion, after which they showed oculomotor performance compatible with gravitational expectation. Thus, concurrence of ocular tracking and manual interception strongly influenced the temporal recruitment of the gravity prior.

## Introduction

Visual information is crucial to interact efficiently with the environment, particularly when surrounding objects are moving. In this case, besides the object’s location, also information about its kinematics and the potential physical interaction with other objects becomes essential. Acquisition of high-definition visual information about the moving target is afforded by eye movements that track its motion, bringing and maintaining its image on the fovea [1–3]. Tracking of a target moving on the fronto-parallel plane is generally achieved by combining saccadic movements, that rapidly shift the gaze to bring the object’s image from the peripheral retina to the fovea with smooth pursuit eye movements (SPEMs), which, by moving the eyes at the same velocity as the visual object, stabilize the retinal projection of the moving object on the fovea [4]. The time required for processing visual information and executing these eye movements, however, introduces potentially significant delays, which must be accounted for to ensure accurate tracking [5]. Predictive mechanisms that anticipate the target future behavior, thus, can play a decisive role to overcome these delays [6,7], becoming more predominant when target information is temporarily missing –e.g., because of a temporary visual occlusion or in case of large gaze shifts [8–11]. Predictive estimates of the target motion when visual motion is prevented by an occluder can be based on signals about the target kinematics immediately before the occlusion [12], integrated with cognitive factors and a priori information derived from past experiences [13–15]. A priori information may include also factors related to the overall naturalness of the visual environment and long-term memory of the dynamic interactions between the target and the surrounding environment, such as bounces against surfaces or deflections [8,16–20]. In this respect, gravity represents a major physical invariant of the Earth environment, imposing ~9.81 m s^-2^ downward acceleration at sea level to free-moving targets and contributing to the perceived naturalness of the environment [21]. Several studies have gathered evidence that an internal model of gravity effects on the objects’ motion may play a role during manual interceptive actions to predictively estimate the time to contact with objects under the effect of gravity [22–26]. For a review, see [27], or during visual search tasks [28] and during the processing of vestibular information [29–31]. This internal model may account for the accurate temporal and spatial estimates driving the interception of free-falling accelerating targets, in spite of the relatively poor sensitivity of the visual system to accelerated motion [32–38]. More recent experimental evidence has extended the notion of the internal model of gravity to the oculomotor control [8,16,18,39,40]. For example, Delle Monache et al. (2015) [41] showed that ocular tracking of trajectories perturbed with effects of altered gravity reflected expectation of gravity effects in a similar fashion to interceptive movements of the same target trajectories. Alike interceptive movements, the relative weight of a priori and sensory information driving eye movements depended strongly on the visual context, since participants seemed to rely more strongly on sensory information when tracking targets against a uniform background, while expectations of gravity effects were more evident when the same target trajectories were tracked in a quasi-realistic pictorial background [39]. However, during periods of transient disappearance of the visual targets, a priori expectations of gravity effects were evident with both visual contexts.

In addition to the subjects’ expectations on the motion of the visual object, another factor that can influence the eye-tracking behavior is the execution of a contingent task. Renowned experiments by Yarbus have shown that, during the mere exploration of a scene, eye movement trajectories relate to the aim of that exploration [42]. Moreover, demanding cognitive tasks influence oculomotor performance, with a clear negative correlation between the complexity of the task and the eye movements accuracy [43–47].

A behavior often associated with the ocular tracking of a moving object is the manual interception. During interceptive actions, eye and hand movements are generally coupled [48] and this coupling does provide an advantage to the interceptive performance, with a clear increase in the interception accuracy [49]. Eye motion generally tends to lead hand movements [41,48,50], preceding the hand motion onset [51]. Corollary signals related to oculomotor predictions when the target motion is predictable may contribute to the planning of the interceptive action, accounting, perhaps, for the improvement of the interceptive performance, even independently from the eye-tracking performance itself [15, 52-54]. Furthermore, control centers for oculomotor and manual interception may share predictive information related to the effects of gravity on the object motion [39,41]. Specifically, Delle Monache and colleagues in an earlier study (2015) [41] examined eye movement patterns during interception of visual targets moving with laws of motion either congruent or not with natural gravity and that could be occluded or not for variable time intervals. Under these experimental conditions, eye movement patterns depended on targets’ laws of motion and visibility, suggesting predictive mechanisms. With occluded targets, better interceptive performance was associated to greater eye tracking accuracy, supporting the idea that precise ocular tracking could provide better target motion predictions for the interceptive response. In this study, however, participants moved their eyes freely, producing idiosyncratic patterns which did not allow to evaluate systematically how the concurrent execution of the manual interceptive task influenced the ocular tracking performance.

In the present study, we took a different approach to investigate more in depth the degree to which ocular tracking performance could be influenced by the concurrent execution of a manual interception and could reflect the use of shared a priori gravity information. Two groups of subjects tracked visual targets, which moved either congruently or not with the effects of gravity over either a pictorial or a neutral background. To enforce predictive mechanisms, targets were occluded for variable time intervals. One group of participants was required to perform only continuous ocular tracking of the visual target, while the other group performed concurrently continuous ocular tracking and manual interception of the visual target. Oculomotor performance was evaluated during two temporal windows related to the target kinematics perturbation and to its occlusion.

We opted for a between-subjects rather than a within-subjects design, because it provided a balanced trade-off between the statistical constraints and the practical feasibility. Between-subjects designs, in fact, could reduce the risk of contamination across conditions and help preserving the independence of task contexts, which is particularly important when tasks differ substantially in structure or content [55]. Conversely, applying a within-subjects design would have increased the experiment duration to about three hours, raising the issue of controlling for fatigue, attention loss, and carryover effects.

We hypothesized that if ocular tracking control mechanisms relied on an expectation of gravity effects, as suggested also by earlier studies, the oculomotor indexes characterizing saccadic and smooth pursuit kinematics would show systematic dependence on the acceleration levels imposed to the visual targets, similar to what reported for the interception of objects under comparable experimental conditions [23,25,26,56]. Conversely, if control of ocular tracking was based on mechanisms relying primarily on sensory information, eye movement kinematics would reflect merely the target speed changes associated to the experimental manipulations of the target acceleration [57–60]. Thus, we tailored an analysis of the ocular tracking responses aimed at dissociating more effectively the effects of target acceleration and speed on the oculomotor behavior, an issue that was not previously accounted for by the earlier studies. With respect to the concurrent execution of the interceptive and ocular tracking task, we hypothesized that it could affect the oculomotor performance by modulating the recruitment of predictive gravity information, reflecting the effective sharing of predictive information by the two control systems.

Overall, our results supported further the notion that both eye tracking and interceptive movements can reflect expectations of gravity effects, rather than the actual target velocity. The effects of gravitational expectations were more evident with the pictorial background, reinforcing the idea that the visual context can facilitate the recruitment of the gravity prior. More remarkably, the concurrence of eye tracking and manual interception appeared to influence the temporal course with which gravity internalized information was engaged, by aligning it to that suggested for similar forms of interceptive action.

## Methods

Sixty-nine volunteers signed informed written consent to participate to the experimental procedures. A priori power analysis yielded a required sample size of 68 participants to detect an effect size f of 0.5 with α = 0.05 and power of 0.9. Participants’ vision was either normal or corrected-to-normal. Subjects were randomly divided into two groups: the first group comprised 37 subjects (20 women, 17 men; mean age 23.88 years ± 2.87 SD); the second group comprised 32 subjects (18 women, 14 men; mean age 24.22 years ± 3.52 SD). Volunteers belonging to the second group –who were required to perform a manual interception task, in addition to the oculomotor task– were tested for handedness by the abbreviated version of the Edinburgh Handedness Inventory [61]. The large majority (n = 25) were right-handed, five were ambidextrous, the remaining two, albeit left-handed, preferred using the right hand to control the computer mouse (25, 5 and 2 respectively). Participants of the first group were not tested for handedness because they performed only the oculomotor task.

Experimental procedures were performed in agreement with the Declaration of Helsinki and approved by the ethics committee of the University of Rome “Tor Vergata” (protocol n. 140.12).

### Visual scenes

Subjects seated 60 cm away from a 22-in. LCD screen (ViewSonic VX2268WM), with their head stabilized by a chin rest. We presented two different visual scenarios, designed by means of the software package Presentation (version 14.9; Neurobehavioral Systems, Berkeley, CA). Scenarios were displayed with a refresh rate of 100 Hz and a spatial resolution of 1,680 * 1,050 pixels.

**Fig 1:**
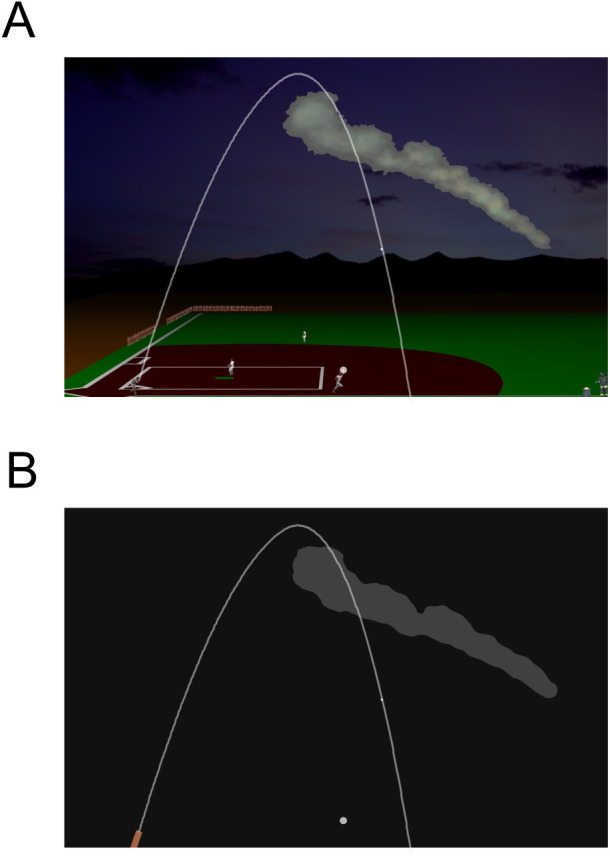
Visual scenes for the Oculo-Manual Task. **A)** In the Pictorial Scenario, the scene represented the fly-ball play of a baseball game. A white ball moved from the bottom left corner of the screen and followed a quasi-parabolic trajectory landing in the right portion of the screen. During its trajectory, the ball was transiently occluded by a smoke-alike cloud positioned in the upper right portion of the scene. Pictorial elements gave participants depth and relative sizes cues. The white semitransparent circle (diameter: 22 pixels) on the glove of the outfielder represented the interception area of the interceptive task. **B)** In the Neutral Scenario, the white target moved on a dark-grey background with no graphic elements except for an orange rectangle on the lower left portion of the screen and an irregularly shaped light-gray geometric element on the upper right area of the scene. The white semitransparent circle had the same initial position as in the Pictorial Scenario. The parabolic white path (representing the trajectory of a target with initial velocity of 25 deg of visual angle, perturbed with 2g 1750 ms before its landing, and occluded for 450 ms) is shown here for illustrative purposes only but never appeared on screen. Visual scenes for the Oculomotor Task were identical but without the white semitransparent circle.

One scenario, the Pictorial Scenario, represented a play from the baseball game and contained quasi-realistic graphic elements and depth cues. A visual target started its motion from a baseball hitter located at the bottom left corner of the baseball field (Fig 1A). During its quasi-parabolic trajectory (see below), the target was transiently occluded by a smoke-like cloud (occluder) positioned, from trial to trial, in different locations on the upper right area of the screen. For the manual interception task (see below), the position of an outfielder, initially located at the lower central portion of the scene, could be controlled by displacing the computer mouse along the horizontal axis. A semitransparent circle (22 pixels diameter, 0.62 deg visual angle) surrounded the outfielder’s right hand to delimit the valid interception area.

The other scenario, the Neutral Scenario, consisted of a dark-grey background (RGB colormap: 17, 17, 17) containing several geometrical elements. An orange rectangle represented the starting position of the target motion and a light gray irregularly-shaped geometric element occluded the target motion like the cloud of the Pictorial Scenario, to which it was matched for mean luminance (RGB colormap: 64, 64, 64; Fig 1B). In this scenario, the moving cursor for intercepting the visual target was a white circle (RGB colormap: 255, 255, 255) identical in size and starting position to the circle surrounding the outfielder’s hand in the Pictorial Scenario.

### Trajectories

**Fig 2:**
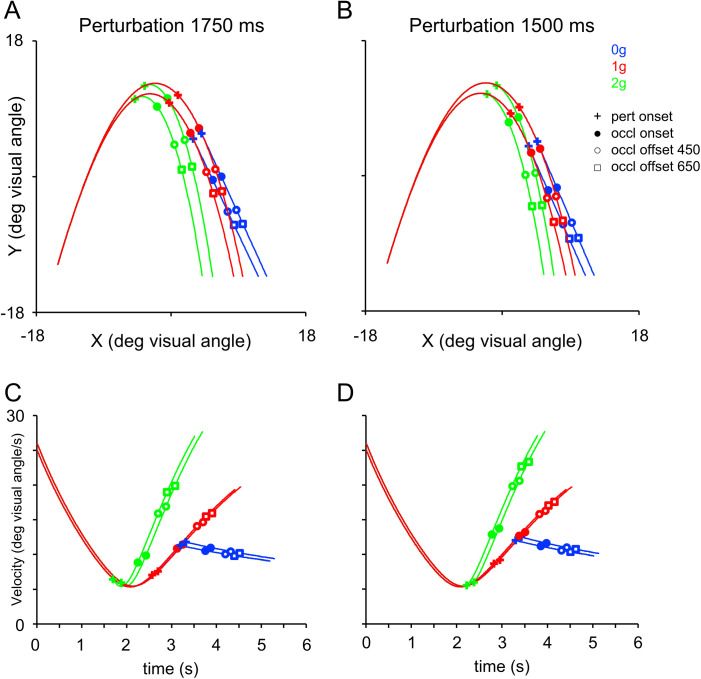
Target trajectories and velocity profiles. The ascending portion of the trajectory was modeled accounting for the effects of Earth gravity (scaled to the graphic elements of the Pictorial Scenario, see Fig 1). The descending portion of the trajectory could either retain the effects of gravity (1g, red) or be perturbed to simulate a micro-gravity (0g, blue) or a hyper-gravity (2g, green) condition. The + symbol indicates the point of the trajectory at which the kinematic perturbation was applied to produce 0g and 2g trajectories, while for unperturbed 1g trajectories it represents the temporal marker for determining the onset of the target occlusion (see the main text for details). 550 ms after the temporal marker of the perturbation, targets were temporarily occluded (filled circles) reappearing 450 ms (open circles) or 650 ms (open squares) later. A) trajectories perturbed 1750 ms before the nominal interception point. B) trajectories perturbed 1500 ms before the nominal interception point. **C)** Velocity profiles of the trajectories perturbed 1750 ms before the nominal interception point. Profiles are shown from the onset of the target motion to nominal interception point. **D)** Velocity profiles of the trajectories perturbed 1500 ms before the nominal interception point. See Table 1 for further details on the target kinematics.

Visual targets (white circles with a diameter of 7 pixels, 0.20 deg visual angle, RGB colormap: 255, 255, 255) followed ballistic trajectories confined to the frontal plane. Targets’ kinematics were in accordance with motion equations described by Brancazio (1985) [62] for a baseball fly ball affected by gravity and air drag see also [23]. Briefly, unperturbed fly-ball trajectories for a projectile were derived from Newtonian mechanics:


$$m\ddot{x}=-F_{D}\text{cos}\vartheta =-F_{D}\left(\frac{\dot{x}}{v}\right)$$ (1)

$$m\ddot{y}=-F_{D}\text{sin}\vartheta =-F_{D}\left(\frac{\dot{y}}{v}\right)-ma$$ (2)

where *F*_*D*_ indicates the aerodynamic drag force vector magnitude (depending on air density, ball cross-sectional area, ball scalar velocity and a drag coefficient, and with its direction being opposite to the velocity vector v), $\dot{x}$ and $\dot{y}$ representing horizontal and vertical components of velocity, $\ddot{x}$ and *ÿ* the horizontal and vertical components of the ball acceleration, m the ball mass, ϑ the ball angle and *a* the target acceleration. We defined the equations of motion for a projectile moving in a two-dimensional field as Eq. 1 and Eq. 2. We then obtained the *x* and *y* coordinates of the ball by integrating Eq. 1 and 2 over time intervals corresponding to each video frame (10.00 ms). Targets’ launch angle was fixed to 73 deg from the horizontal, while initial velocities could assume two possible values, instrumental to increase the variability in the experimental conditions (Fig 2 and Table 1). The ascending portion of the trajectory accounted for the effects of the gravitational acceleration (g = 9.81 ms^-2^) scaled to the graphic elements of the Pictorial Scenario, whereas the descending portion could be either perturbed or not with altered gravity effects. Perturbations occurred at temporal markers of 1750 ms or 1500 ms before target landing: after these temporal markers, in 1/3 of the trials, targets retained gravity effects (1g conditions), while in the remaining 2/3 they could either assume constant velocity motion (0g conditions, 1/3 of the total number of trials) or increase instantly their constant acceleration to 2g (2g conditions, 1/3 of the total number of trials). Then, 550 ms after the perturbation marker, the target disappeared behind the occluder (the smoke-like cloud or the light-gray geometric element in the Pictorial and in the Neutral Scenario, respectively) to reappear either 450 ms or 650 ms later. Depending on the combination of perturbation and occlusion intervals, the target became visible again before landing for a time interval comprised between 300 and 750 ms. Alike the manipulations of the target initial velocity, the two perturbation intervals and the two occlusion intervals were introduced merely to increase the numerosity of the experimental conditions (i.e., increase the number of different trajectory types) and prevent that subjects’ oculomotor performance could reflect categorization of a reduced number of target trajectories. Thus, overall, 24 experimental conditions were defined by combining 3 laws of motion (0g, 1g, 2g), 2 perturbation intervals (1750 ms, 1500 ms), 2 occlusion durations (450 ms, 650 ms) and 2 initial velocities. For each scenario, we presented 8 repetitions of each experimental condition (192 trials, overall), distributed pseudo-randomly. Trials with the two different scenarios were performed in separate blocks, with a 10-minute resting break between them. The order of the two scenarios was counterbalanced across subjects (Pictorial first and Neutral first).

**Table 1:** Kinematic values of target trajectories. To directly compare oculomotor indexes across different occlusion durations, we computed them between 100 ms and 450 ms after occlusion onset. Therefore, in the mean *occlusion* window velocity column values are identical between conditions with identical combinations of law of motion, perturbation and initial velocity. The parameters represented in each column are defined as follows. *Gravity Level* (*g* = 9.81 m/s^2^); *Pert onset*: perturbation temporal marker; *Occl dur*: occlusion duration; *Initial vel*: initial target velocity; *Dur pre-pert*: duration of the pre-perturbation marker portion of the target trajectory; *Pert onset vel*: target velocity at the perturbation temporal marker; *Mean pert window vel*: mean target velocity in the *perturbation* window; *Occl onset vel*: target velocity at the occlusion onset; *Mean occl window vel*: mean target velocity in the *occlusion* window; *Dist occl onset – occl offset*: distance between the occlusion onset and the occlusion offset; *Dist launch – interception*: distance between the beginning of the target trajectory and the nominal interception point.

Gravity Level	Pert onset (ms)	Occl dur (ms)	Initial vel (deg vis angle/s)	Dur pre- pert (ms)	Pert onset vel (deg vis angle/s)	Mean pert window vel (deg vis angle/s)	Occl onset vel (deg vis angle/s)	Dist launch – occl onset (deg vis angle)	Mean occl window vel (deg vis angle/s)	Dist occl onset – occl offset (deg vis angle)	Dist launch -interception (deg vis angle)
0*g*	1750	650	25.0	3190	11.3	10.9	10.6	23.5	10.3	6.6	26.8
			26.0	3310	11.8	11.3	11.0	24.7	10.7	6.9	27.9
		450	25.0	3190	11.3	10.9	10.6	23.5	10.3	4.6	26.8
			26.0	3310	11.8	11.3	11.0	24.7	10.7	4.8	27.9
	1500	650	25.0	3290	12.0	11.5	11.2	23.2	10.9	7.0	26.1
			26.0	3410	12.5	12.0	11.7	24.5	11.3	7.3	27.2
		450	25.0	3290	12.0	11.5	11.2	23.2	10.9	4.9	26.1
			26.0	3410	12.5	12.0	11.7	24.5	11.3	5.1	27.2
1*g*	1750	650	25.0	2560	7.0	9.2	10.8	24.9	12.8	8.6	23.5
			26.0	2690	7.5	9.8	11.4	26.1	13.4	8.9	24.8
		450	25.0	2560	7.0	9.2	10.8	24.9	12.8	5.6	23.5
			26.0	2690	7.5	9.8	11.4	26.1	13.4	5.9	24.8
	1500	650	25.0	2810	8.6	11.0	12.6	24	14.6	9.7	23.5
			26.0	2940	9.2	11.6	13.2	25.2	15.1	10.0	24.8
		450	25.0	2810	8.6	11.0	12.6	24	14.6	6.4	23.5
			26.0	2940	9.2	11.6	13.2	25.2	15.1	6.6	24.8
2*g*	1750	650	25.0	1690	6.5	6.4	8.7	24.8	13.0	9.0	19.3
			26.0	1870	6.0	7.0	9.7	26.4	14.0	9.6	20.7
		450	25.0	1690	6.5	6.4	8.7	24.8	13.0	5.5	19.3
			26.0	3190	6.0	7.0	9.7	26.4	14.0	6.0	20.7
	1500	650	25.0	3310	5.5	9.3	12.8	24.6	17.1	11.6	20.6
			26.0	3190	5.9	10.1	13.6	26	17.9	12.1	22
		450	25.0	3310	5.5	9.3	12.8	24.6	17.1	7.4	20.6
			26.0	3290	5.9	10.1	13.6	26	17.9	7.7	22

### Behavioral tasks

As mentioned above, subjects were assigned randomly to one of two groups. Subjects assigned to the first group were required to track continuously with their eyes the visual target throughout its trajectory, including the visually occluded portion (Oculomotor Task). Subjects assigned to the second group performed concurrently continuous ocular tracking of the entire target trajectory and its manual interception (Oculo-Manual Task). That is, while both subject groups tracked the visual target continuously, the second group, at the same time, was also engaged in the manual interception of the target. For this purpose, subjects assigned to the second group used a gaming mouse (Razer Copperhead, San Francisco, CA) to displace horizontally a cursor on the screen (i.e., the outfielder’s hand surrounded by the white circle in the Pictorial Scenario or the white circle in the Neutral Scenario) from its initial central position towards the target landing location in the bottom right corner of the scene. They indicated the interception of the visual target with the white semitransparent circle by pressing the left mouse button. Subjects were not provided with feedback of the interceptive performance, as the target kept moving towards the lower border of the visual scene disappearing thereafter. Because continuous ocular tracking of the target was imposed for both the Oculomotor Task and the Oculo-Manual Task, we were able to compare directly the ocular tracking performance between the two subject groups by using the same oculomotor indexes. The manual interception task was identical to that described by previous studies of our group [23,41], but the oculomotor requirements of the participants were profoundly different: while in the current experiments continuous ocular tracking was enforced, in the previous studies, subjects’ oculomotor behavior was not constrained, but they could move their eyes freely throughout the visual scene following patterns instrumental to the manual interception of the visual target.

### Data acquisition

Subjects’ binocular eye movements were recorded with an EyeLink 1000 tracker system at a sampling frequency of 500 Hz (SR Research, Ontario, Canada). The eye tracker was calibrated at the beginning of each experimental session and every 32 trials with a 9-point calibration grid, while drift corrections were applied every 8 trials. Thus, 9-point calibrations were performed, on average, every 232.6 s ± 1.2 SEM and 264.3 s ± 4.3 SEM for participants to the Oculomotor Task and the Oculo-Manual Task, respectively, while drift corrections were performed, on average, every 46.8 s ± 0.1 SEM and 55.9 s ± 0.9 SEM for participants to the Oculomotor Task and the Oculo-Manual Task, respectively. The slightly larger mean values for the Oculo-Manual Task participants reflect merely the fact that, for this task, a new trial started only after the participant repositioned the mouse cursor at its initial centered position, whereas for the Oculomotor Task a new trial started automatically after the end of the previous one. The mouse cursor position during the Oculo-Manual Task was sampled at 200 Hz while button presses were sampled at 1 kHz.

### Eye movement analysis

**Fig 3:**
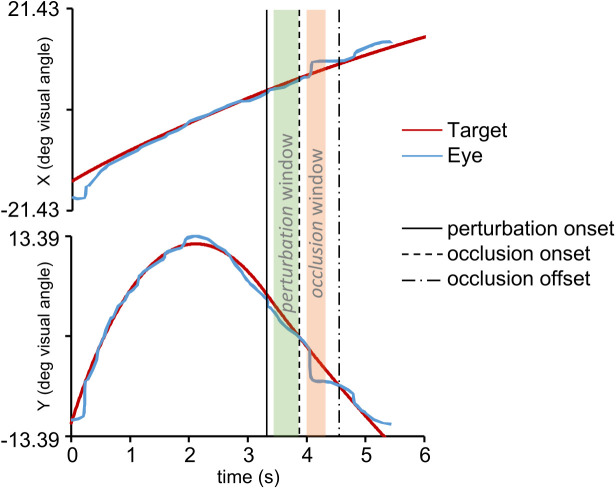
Definition of the *perturbation* and *occlusion* windows for the analysis of the oculomotor behavior. Horizontal and vertical monocular eye-traces (blue) and target trajectory components (red) are illustrated. The vertical solid line represents the perturbation temporal marker and the green semitransparent rectangle the *perturbation* window. Similarly, the dashed vertical line represents the occlusion onset and the orange semitransparent rectangle the *occlusion* window. Finally, the dash-dotted vertical line represents the reappearance of the target.

Eye tracker signals from both eyes were analyzed preliminarily by means of a custom-made MATLAB (2022a; Mathworks, USA) script to detect blinking artifacts or missing data in the eye position traces. With this preliminary screening, we identified 299 (1.1%) out of 26,496 trials (the total number of trials in the experiment series resulting from 384 trials for each experiment multiplied by 69 subjects) that had to be discarded because signals from both eyes were not usable or missing. For 6,555/26,496 trials (24.7%), only signals from one eye were not usable or missing, but we retained data from the unaffected eye. For the remaining 19,642/26,496 trials with valid binocular eye traces, we computed the cyclopean eye trajectory by averaging the eye traces from the two eyes. Averaging the two eye traces, which was instrumental to improve further the signal to noise ratio, was justified by a preliminary analysis showing that signals from the two eyes were very strongly correlated (r > 0.97 across trials, p < 0.001). Thus, overall, data from 26,197/26,496 trials (98.9%), obtained either from the spared trace of one eye or by averaging the signals from both eyes, were used for further analyses. Notably, the fraction of valid trials identified after the preliminary analysis was equal between the two subject groups (14,052/14,208, 98,9% for participants performing the Oculomotor Task and 12,145/12,288, 98,9% for participants performing the Oculo-Manual Task). Valid eye-tracker signals were numerically differentiated and filtered with a zero-lag 2nd-order low-pass Butterworth filter (cutoff frequency of 40 Hz, see [39, 41, 63]). We evaluated subjects’ eye-tracking behavior during the two temporal windows illustrated in Fig 3: 1) the *perturbation* window (highlighted in *green*), beginning 100 ms after the perturbation temporal marker and ending at the visual occlusion onset 450 ms later; 2) the *occlusion* window (highlighted in *orange*), starting 100 ms after the target occlusion and ending 350 ms later. We did not consider the first 100 ms of data after the perturbation and occlusion events to take into account the visuomotor delays occurring between the incoming visual information and the oculomotor response [39,64]. The duration of the *occlusion* window was chosen to include the entire occluded portion of the trajectory for the two shortest occlusion durations and equalize the duration of the temporal window under analysis among trajectories with different occlusion durations.

Since ocular tracking included smooth pursuit bouts alternated with saccadic movements, we derived from the eye traces several indexes related to the saccadic and the smooth pursuit movements. Saccadic movements were detected by using combined eye-velocity (> 30 deg visual angle/s) and eye-acceleration (> 2,000 deg visual angle/s^2^) thresholds [39,41,65]. However, saccadic movements that produced post saccadic eye-target distances larger than 4 deg of visual angle were discarded, because they diverted much the gaze from the target trajectory, thus not complying with the specific requirement of maintaining continuously the eyes on target [39].

From the valid saccades during the *perturbation* and the *occlusion* windows we extracted two indexes: the post saccadic error and the saccadic τ. The post saccadic error was defined as the distance in visual degrees between the eye position after a saccade and the target, averaged among the first 8 ms (4 samples) after the end of a saccade [39]. The saccadic τ was defined as the post saccadic lag/lead of the eye relative to the visual target (positive value = eye lags the target; negative value = eye leads the target).

Smooth pursuit bouts were identified from eye traces after saccades removal, as time epochs with mean eye-to-target distance within 4 deg of visual angle and mean ratio between eye and visual target velocities comprised between 0.25 and 1.80 [39]. Moreover, we considered only smooth pursuit bouts lasting at least 250 ms in the *perturbation* window and at least 100 ms in the *occlusion* window. Note that the rather high value of 4 deg of visual angle was to comply with the maximum value of post saccadic error allowed for catch-up saccades to be on the target [39]. These more compliant boundaries account for increased ocular tracking variability during visual occlusion [66–68], such as during the *occlusion* window of the present experiments. Consistency analysis between 4 and 2 deg of visual angle datasets confirmed preserved within-subjects patterns across oculomotor indexes, with moderate-to-high Intraclass Correlation Coefficients (ICCs) across the factors used as “within subjects” predictors in the full factorial ANOVA models described below (Gravity Level: 0.40–0.96; Visual Scenario: 0.46–0.95; *Perturbation*/ *Occlusion* Window: 0.60–0.96/0.35–0.94), overlapping bootstrapped effect sizes (|Δd| = 0.01–0.32), and Two One-Sided Tests (TOST) equivalence (effects within ±0.2; all p < 0.001 except upper bound for saccadic τ) consistent with robustness of the effects. The velocity ratio thresholds were defined based on the target velocity ranges of the trajectories from a minimum of 5.4 (2g *perturbation* window) to a maximum of 23.2 deg of visual angle/s (see Fig 2C-D).

From the smooth pursuit bouts we derived the time-lag SPEM τ and the SPEM gain of the pursuit as defined by Mrotek and Soetching (2007) [48]. See also [39,41]. Briefly, SPEM τ is the lag/lead in milliseconds of the eye relative to the visual target, with positive and negative values indicating that eye movements lagged or were ahead of the target, respectively. The SPEM gain measures the projection of the eye velocity vector on the target velocity vector and, thus, it is a non-dimensional variable. By this definition, a gain of 1 occurs if the eyes move at the same speed and in the same direction as the target, whereas if the eyes move at the target speed but perpendicularly, or if they are stationary (i.e., the subject is fixating) the gain is equal to 0. τ and gain values were computed for each 2 ms data samples and then averaged over the pursuit bout.

### Manual interception analysis

To characterize the interceptive behavior in the Oculo-Manual Task, we defined three indexes related to the button press responses and to the mouse displacements: the timing error, the positional error and the velocity of the mouse cursor at the time of the button press (velocity at response). The timing error was defined as the temporal difference in milliseconds between the time of the button press and the nominal interception time (i.e., the time at which the target reached the same height in the visual scene as the center of the white circle, which delimited the valid interception area). Negative and positive values indicated anticipated (early) and delayed (late) interceptive responses, respectively. The positional error represented the distance in visual angle between the horizontal location of the white circle at the time of the interceptive response and the horizontal position at which the target trajectory reached the same height as the center of the white circle. Negative values indicated underestimation of the target landing locations whereas positive values indicated their overestimation. The velocity at response was measured in visual angle sec^-1^ with positive and negative values indicating, respectively, rightward and leftward ongoing displacement of the outfielder at the time of the button press response. We considered this parameter to be indicative of whether subjects were performing corrective movements at the time of the response either in the direction of the target motion (denoting underestimation) or in the opposite direction (denoting overestimation). Instead, cursor velocity values close to zero would indicate that subjects had made definite estimates of the target landing location at the time of the button press.

### Statistical analyses on oculomotor indexes

#### Full factorial ANOVA models.

Given that the main focus of the study was assessing the effects of the naturalness of the visual scene and of the concurrent execution of the interceptive task on the ocular tracking, as first approach, we averaged the index values across trials with different initial velocities, perturbation durations and occlusion durations, namely, the manipulations introduced merely to increase the number of different experimental conditions (see above the description of the target trajectories). Thus, for each oculomotor index we pooled, across experimental subjects, the mean values for the reduced number of experimental conditions resulting from collapsing data with respect to target initial velocity, perturbation and occlusion interval, and submitted the resulting datasets to full factorial mixed ANOVA models with the following predictors: Window (W, 2 levels: *perturbation* | *occlusion*), visual Scenario (S, 2 levels: Pictorial | Neutral), Gravity Level (GL, 3 levels: 0g | 1g | 2g) as *within-subjects* factors; Scenario Order (SO, 2 levels: Pictorial first | Neutral first), and behavioral Task (T, 2 levels: Oculomotor | Oculo-Manual) as *between-subjects* factors. The significance cut-off for main and interaction effects was set to p < 0.01, Greenhouse-Geisser corrected. Moreover, to facilitate interpretation of the significant ANOVA factors, we performed pairwise comparisons between factor levels by using either paired t-tests or two-sample t-tests for dependent or independent measures, respectively (significance cut-off p < 0.01, Bonferroni corrected for multiple comparisons). Consistent with current recommendations [69], we applied restrictive significance cut-offs for the ANOVA effects and the pairwise comparisons to reduce the potential impact of Type I errors, considering also the presence of between subject factors and their interactions in the ANOVA models that could be prone to effects of intergroup variability.

#### Analysis of oculomotor indexes with respect to the target velocity.

Because of the particular design of the ballistic trajectories, where acceleration perturbations and visual occlusions occurred at different locations in the trajectory path, mean velocities during the *perturbation* and *occlusion* windows were different across gravity levels (see Table 1). This, inevitably, introduced a potential confound, in that gravity levels might be uniquely associated to the mean target velocities, particularly when collapsing data with respect to the target initial velocity and the perturbation temporal marker. Such experimental confound would make it difficult to attribute any changes in the oculomotor indexes unequivocally to either the target acceleration or velocity.

To address this issue, we compared the mean values of the oculomotor indexes in response to each trajectory type (i.e., without collapsing data for perturbation or initial velocity) under two complementary conditions: 1) identical gravity levels but different target velocities, and 2) comparable target velocities but different gravity levels. For these analyses we considered the mean target velocity during the *perturbation* window and, for the *occlusion* window, the target velocity at the occlusion onset. We adopted a cut-off criterion of less than 10% velocity difference between conditions with different gravity levels based on previous studies reporting that, under foveal vision, velocities are perceived as similar if they differ less than 6% [70,71], a value that increases with aperture [72] and attentional [73] factors. According to this criterion, we identified 13 possible comparisons for the *perturbation* window and 14 for the *occlusion* window. By the same token, we considered two conditions with the same gravity level having different velocity if the velocity difference was greater than 10%. This procedure identified 9 possible comparisons for the *perturbation* window and 10 for the *occlusion* window.

Then, we carried out paired t-tests between mean values of the oculomotor indexes separately for Task, Window and Scenario conditions. If a paired t-test between conditions with comparable velocity but different gravity level was significant (p < 0.01 Bonferroni corrected) we attributed the effect to the manipulation of the gravity level. Conversely, if a statistically significant paired t-test occurred between conditions with the same gravity level but different velocity, we attributed the effect to the target velocity. Finally, we compared the rates of statistically significant comparisons between similar and different gravity levels conditions with χ^2^ statistics to assess the degree to which differences in oculomotor indexes reflected gravity level or target velocity changes.

#### Analysis of the temporal trends of the oculomotor indexes.

Besides evaluating the general effects of the experimental manipulations on the oculomotor performance, we also aimed at characterizing potential adaptive responses to the experimental conditions. For this purpose, we fitted double exponential curves to the time series of the mean oculomotor indexes among subjects for all experimental conditions. We evaluated the statistical significance of the double-exponential fits by their R^2^ and associated p-values, setting the cut-off of p < 0.01.

### Statistical analyses on interception indexes

For the analysis of the indexes related to the interceptive performance (Timing error, Positional error, Velocity at response) we used the same approach applied to the oculomotor indexes by compiling datasets with the reduced set of experimental conditions. These datasets, resulting from pooling the mean values of the manual interception indexes across participants to the Oculo-Manual task, were submitted to full factorial mixed ANOVA models with visual Scenario (2 levels: Pictorial | Neutral) and Gravity Level (3 levels: 0g | 1g | 2g) as *within-subjects* factors, and Scenario Order (Pictorial first | Neutral first) as *between-subjects* factor. The significance cut-off for main and interaction effects was set to p < 0.01, Greenhouse-Geisser corrected. We compared differences between levels of the significant factors by means of paired t-tests or two-sample t-tests for dependent or independent measures respectively (significance cut-off p < 0.01, Bonferroni corrected for multiple comparisons).

## Result

We studied the effects on the ocular tracking performance of manipulating the degree of realism of the visual scene and of the concurrent execution of a manual interception. Separate groups of participants performed either only ocular tracking of the moving target or concurrent ocular tracking and manual interception of the visual target. Eye tracking performance was assessed for two distinct temporal windows: the *perturbation* window, related to the manipulation of the target gravity acceleration; the *occlusion* window during which vision of the target motion was prevented.

### Ocular tracking performance

**Fig 4:**
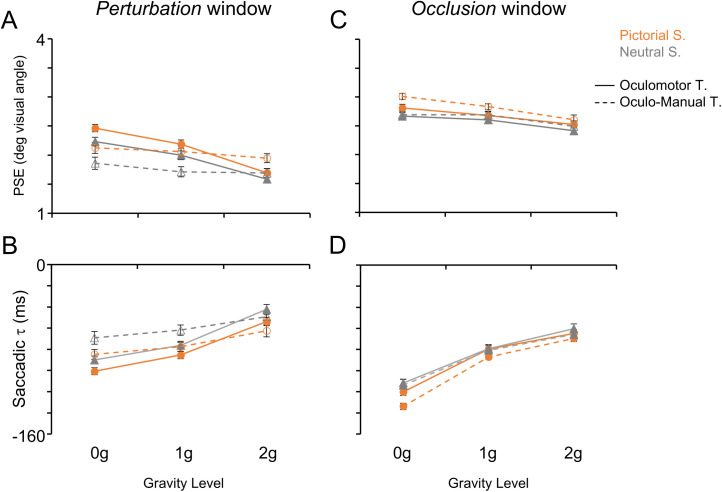
Saccadic performance during the *perturbation* (A-B) and *occlusion* (C-D) windows. Each panel shows the mean (± SEM) oculomotor parameters measured during ocular tracking of the visual targets with either the Pictorial Scenario (orange and circle markers) or the Neutral Scenario (dark grey and triangular markers). Data collected from subjects performing the Oculomotor Task are denoted by filled markers and solid lines, whereas those collected from subjects performing the Oculo-Manual Task are denoted by open markers and dashed lines and are grouped across the three law of motion values (0g, 1g and 2g). A, **C)** PSE, post saccadic error. B, **D)** Saccadic τ.

**Fig 5:**
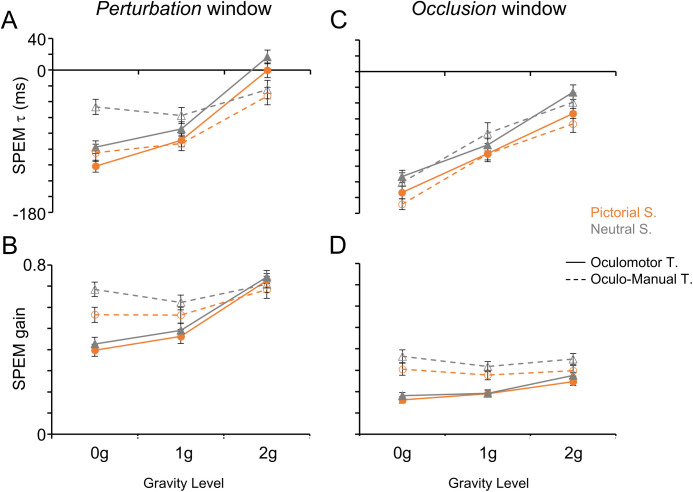
Smooth pursuit performance during the *perturbation* (A-B) and *occlusion* (C-D) windows. Same layout as Fig 4. A, **C)** SPEM τ. B, **D)** SPEM gain.

In general, ocular tracking indexes were strongly influenced by factors related to the availability of visual information about the target (i.e., *perturbation* and *occlusion* windows), to the overall realism of the visual scene (i.e., the gravity level and the type of visual scenario), and to the type of task performed by participants (see Fig 4 and Table 2 for saccadic indexes, and Fig 5 and Table 3 for SPEM indexes).

**Table 2:** Results of repeated measures ANOVA on indexes characterizing saccadic eye movements (in bold p < 0.010, Greenhouse-Geisser corrected). For clarity, only the main effects and the statistically significant interaction effects are hereby reported. The full table is reported in the Supplementary material as Table 1. Although the p-values reported are Greenhouse-Geisser corrected, to simplify the layout of the table and improve its readability, degrees of freedom (dof) are reported uncorrected, once for all the ANOVA tests. W, Window; S, Scenario; GL, Gravity Level; SO, Scenario Order; T, Task; PSE, post saccadic error.

		PSE			Saccadic τ		
Factor	dof	F	p	η^2^	F	p	η^2^
W	1,65	215.7	**< 0.001**	0.77	45.9	**< 0.001**	0.41
S	1,65	55.2	**< 0.001**	0.46	53.1	**< 0.001**	0.45
GL	2,130	85.9	**< 0.001**	0.57	297.0	**< 0.001**	0.82
SO	1,65	0.3	0.599	< 0.01	0.4	0.530	0.01
T	1,65	< 0.1	0.857	< 0.01	< 0.1	0.856	< 0.01
W*GL	2,130	5.1	**0.008**	0.07	60.2	**< 0.001**	0.48
GL*T	2,130	10.5	**< 0.001**	0.14	5.8	**0.009**	0.08
W*GL*T	2,130	19.1	**< 0.001**	0.23	16.1	**< 0.001**	0.20

**Table 3:** Results of repeated measures ANOVA on indexes characterizing SPEMs (in bold p < 0.010, Greenhouse-Geisser corrected). For clarity, only the main effects and the statistically significant interaction effects are hereby reported. The full table is reported in the Supplementary material as Table 2. Although the p-values reported are Greenhouse-Geisser corrected, to simplify the layout of the table and improve its readability, degrees of freedom (dof) are reported uncorrected, once for all the ANOVA tests. W, Window; S, Scenario; GL, Gravity Level; SO, Scenario Order; T, Task.

		SPEM τ			SPEM gain		
Factor	dof	F	p	η^2^	F	p	η^2^
W	1,65	37.6	**< 0.001**	0.37	393.6	**< 0.001**	0.86
S	1,65	70.0	**< 0.001**	0.52	27.9	**< 0.001**	0.30
GL	2,130	283.3	**< 0.001**	0.81	91.8	**< 0.001**	0.58
SO	1,65	0.2	0.666	< 0.01	0.1	0.779	< 0.01
T	1,65	0.1	0.792	< 0.01	10.7	**0.002**	0.14
W*GL	2,130	18.3	**< 0.001**	0.22	49.5	**< 0.001**	0.43
GL*T	2,130	12.6	**< 0.001**	0.16	39.2	**< 0.001**	0.38
W*S*GL	2,130	6.3	**0.004**	0.09	7.8	**0.001**	0.11
W*GL*T	2,130	14.2	**< 0.001**	0.18	10.8	**< 0.001**	0.14

The availability of visual information about the target motion affected significantly both saccadic and SPEM indexes. Specifically, we found that post saccadic errors were higher in the *occlusion* than in the *perturbation* windows. Moreover, in the absence of target visual information, saccadic τ and SPEM τ values were more negative, while SPEM gains were lower. This denoted a larger anticipation of the target position, even by way of predictive saccades, accompanied by lower eye velocities. The lower SPEM gains go along also with the general observation that, in the absence of target visual information, SPEM gains decrease consistently [53,74,75].

Perturbations of the target acceleration affected significantly both saccadic and smooth pursuit movements, with ocular tracking indexes showing generally monotonic relationships with respect to the gravity level, which accounted for the highly significant main effects of this predictor (Tables 2-3). Specifically, post saccadic errors were highest and both saccadic and smooth pursuit time leads were largest during 0g than 1g and 2g trials (see Figs 4-5 and Tables 2-3). In fact, paired t-tests evaluating differences between gravity levels for post saccadic error, saccadic τ, SPEM τ and SPEM gain values were all statistically significant (p < 0.001). These differences reflected trends of monotonically decreasing values of post saccadic error and monotonically increasing saccadic τ, SPEM τ and SPEM gain values with increasing gravity levels.

In addition to the target acceleration, the presence of pictorial realistic elements in the visual scene strongly influenced the oculomotor behavior (Figs 4-5). Specifically, higher post saccadic errors and more anticipatory (i.e., negative) saccadic and SPEM τ values were observed in the Pictorial than in the Neutral scenarios (Tables 2-3). Conversely, higher SPEM gains were observed in the Neutral compared to the Pictorial scenarios.

SPEM gains were also influenced by whether the Oculomotor or the Oculo-Manual Task was being performed (main effect of Task in Tables 2-3). Indeed, SPEM gains were higher (Fig 5) in subjects performing the Oculo-Manual than in those performing the Oculomotor Task.

A statistically significant two-way interaction effect of Gravity Level*Task was observed in several ocular tracking indexes, which showed steeper monotonic trends with the gravity level during the Oculomotor compared to the Oculo-Manual task. Indeed, paired t-tests showed that pairwise differences between acceleration levels were stronger in the Oculomotor Task for the post saccadic error, saccadic τ, SPEM τ and SPEM gain (see Table 4 for pairwise comparisons across gravity levels). Moreover, with respect to the SPEM gain values, we observed significantly larger values in the Oculo-Manual than in the Oculomotor Task in 0g and 1g trials (t_(56.9)_ = 5.4, p < 0.001 and t_(63.0)_ = 3.3, p = 0.001 for 0g and 1g trials, respectively).

**Table 4:** Pairwise post-hoc comparisons (paired t-tests) for oculomotor indexes with significant two-way interaction Gravity Level*Task (p-values are reported Bonferroni corrected). Positive t values denote greater values in 0g than 1g trials, 0g than 2g trials and in 1g than 2g trials, vice versa for negative values.

		Oculomotor Task		Oculo-Manual Task	
Index	Comparison	t_(36)_	p	t_(31)_	p
**Post saccadic error**	0g vs. 1g	5.6	**< 0.001**	2.4	0.065
	0g vs. 2g	11.2	**< 0.001**	4.8	**< 0.001**
	1g vs. 2g	9.7	**< 0.001**	3.6	**0.003**
**Saccadic τ**	0g vs. 1g	−13.2	**< 0.001**	−8.5	**< 0.001**
	0g vs. 2g	−18.8	**< 0.001**	−10.1	**< 0.001**
	1g vs. 2g	−18.9	**< 0.001**	−6.1	**< 0.001**
**SPEM τ**	0g vs. 1g	−9.5	**< 0.001**	−7.3	**< 0.001**
	0g vs. 2g	−17.6	**< 0.001**	−10.2	**< 0.001**
	1g vs. 2g	−15.4	**< 0.001**	−7.4	**< 0.001**
**SPEM gain**	0g vs. 1g	−5.1	**< 0.001**	2.6	0.045
	0g vs. 2g	−13.8	**< 0.001**	−1.4	0.492
	1g vs. 2g	−13.7	**< 0.001**	−5.3	**< 0.001**

A significant effect of the two-way interaction Window*Gravity Level was also observed with all oculomotor indexes. In particular, post saccadic errors, saccadic and SPEM τ exhibited similar monotonic trends between *perturbation* and *occlusion* windows, with larger errors and higher time leads in 0g than in 1g and in 1g than in 2g trials (p ≤ 0.006 in the *perturbation* window, p < 0.001 in the *occlusion* window). However, post saccadic errors were higher and SPEM gains were lower in the *occlusion* than in the *perturbation* window for all Gravity levels (all p < 0.001), while saccadic and SPEM τ showed larger anticipations in the *occlusion* than in the *perturbation* window only with perturbed 0g and 2g trajectories (p < 0.001) but not with unperturbed 1g (p ≥ 0.020). SPEM gains were larger in the *perturbation* than in the *occlusion* window for all target accelerations (all p < 0.001), with comparable values observed in 0g and 1g trials (p ≥ 0.393 in both windows) and larger values in 2g than in 1g trials (all p < 0.001).

The three-way interaction Window*Gravity Level*Task was statistically significant for all oculomotor indexes. In general, this interaction effect denoted differences between subject groups performing either the Oculomotor or the Oculo-Manual Task with respect to the dependence of the oculomotor behavior on the gravity level during the *perturbation* compared to the *occlusion* window. In essence, during the *perturbation* window, the oculomotor indexes of subjects performing the Oculo-Manual Task showed a significantly weaker dependence on the Gravity Level than those of subjects performing the Oculomotor Task, whereas during the *occlusion* window differences between the two groups of subjects with respect to the effects of Gravity Level were much more subtle. We quantified further the nature of this interaction effect for each oculomotor index by analyzing the pairwise differences across the three predictor levels.

For the post saccadic errors (Fig 4 and Table 2) the interaction was explained by: 1) during the *perturbation* window a monotonically decreasing trend of post saccadic errors with gravity level was evident in subjects performing the Oculomotor Task (all p < 0.001), but not in those performing the Oculo-Manual Task (p = 0.279 and p = 0.651 for 0g vs. 1g and 1g vs. 2g trials, respectively), whereas during the *occlusion* window these trends were comparable between tasks (values did not differ between 0g and 1g trials (p ≥ 0.062) but were significantly lower in 1g than in 2g trials (p ≤ 0.002)); 2) larger error values were observed in the *occlusion* window for all Gravity Levels and Tasks (all pairwise comparisons: p < 0.001); 3) during the *perturbation* window, larger post saccadic error values were made in response to 0g trials in the Oculomotor Task compared to the Oculo-Manual Task (t_(60.3)_ = 3.4, p = 0.001).

For the saccadic τ (Fig 4 and Table 2), the interaction effect was accounted for by: 1) a decreasing monotonic trend with gravity level was observed in both windows with the Oculomotor Task (all p < 0.001) but only in the *occlusion* window with the Oculo-Manual Task (all p < 0.001); 2) larger time leads were observed in the *occlusion* window in response to 0g and 2g trials with the Oculomotor Task (p < 0.001) and in response to 0g and 1g trials with the Oculo-Manual Task (p ≤ 0.004), whereas no differences between *perturbation* and *occlusion* windows were observed in response to 1g trials with the Oculomotor Task (p = 0.697) and in response to 2g trials with the Oculo-Manual Task (p = 0.025); 3) during the *perturbation* window, saccadic τ values were more negative with the Oculomotor than with the Oculo-Manual Task only in response to 0g trials (p = 0.001).

For SPEM τ values (Fig 5 and Table 3) changes accounting for the Window*Gravity Level*Task interaction, were rather similar to those reported for the saccadic τ. In fact, pairwise comparisons showed: 1) monotonic trends with gravity level were evident during both windows with the Oculomotor Task (all pairwise tests between acceleration levels: p < 0.001) and during the *occlusion* window of the Oculo-Manual Task (all pairwise tests: p < 0.001), while with the Oculo-Manual Task, during the *perturbation* window, SPEM τ values in response to 0g and 1g trials were more negative than in response to 2g trials (p < 0.001 in both cases), and comparable to those in response to 0g trials (p = 1.000); 2) larger time leads during the *occlusion* window in response to 0g trials with both tasks (p < 0.001) and to 2g trials with the Oculomotor Task (p < 0.001), whereas no differences between windows were observed in response to 1g trials with both tasks (p ≥ 0.046) and to 2g trials with the Oculo-Manual Task (p = 0.092); 3) SPEM τ values exhibited greater time leads with the Oculomotor compared to the Oculo-Manual Task during the *perturbation* window in response to 0g (p = 0.004) and 2g (p = 0.008) trials.

For SPEM gains (Fig 5 and Table 3) the significant Window*Gravity Level*Task interaction was mostly related to the lower gains, regardless of the task and of the gravity level, observed during the *occlusion* compared to the *perturbation* window (all pairwise tests between windows: p < 0.001).

The three-way interaction effect Window*Scenario*Gravity Level was significant for both SPEM indexes (Fig 5 and Table 3). During the *perturbation* window SPEM time leads were larger when tracking 0g and 1g trajectories with the Pictorial than with the Neutral Scenario (both pairwise tests between scenarios: p < 0.001), whereas during the *occlusion* window SPEM τ differences between scenarios occurred with all trajectory types (all pairwise tests between scenarios: p < 0.001). Moreover, while a decreasing monotonic trend of SPEM τ values with gravity level was observed with both scenarios during the *occlusion* window (all pairwise tests between gravity levels: p < 0.001), during the *perturbation* window the same trend was observed only with the Pictorial Scenario (all pairwise tests between gravity levels during the *perturbation* window: p < 0.001), as with the Neutral Scenario significant differences occurred only between 1g and 2g trials (p < 0.001).

For SPEM gains, the significant three-way interaction Window*Scenario*Gravity Level was explained by the larger values observed in response to 2g than to 1g trials during both windows and with both scenarios (all p < 0.001). Moreover, during the *perturbation* window, gain values were larger with the Neutral compared to the Pictorial Scenario in response to 0g and 1g trials (all pairwise tests between scenarios p ≤ 0.002), while during the *occlusion* window higher gains with the Neutral Scenario were observed in response to 0g and 2g trials (all pairwise tests between scenarios: p < 0.001).

### Oculomotor indexes and target velocity

**Fig 6:**
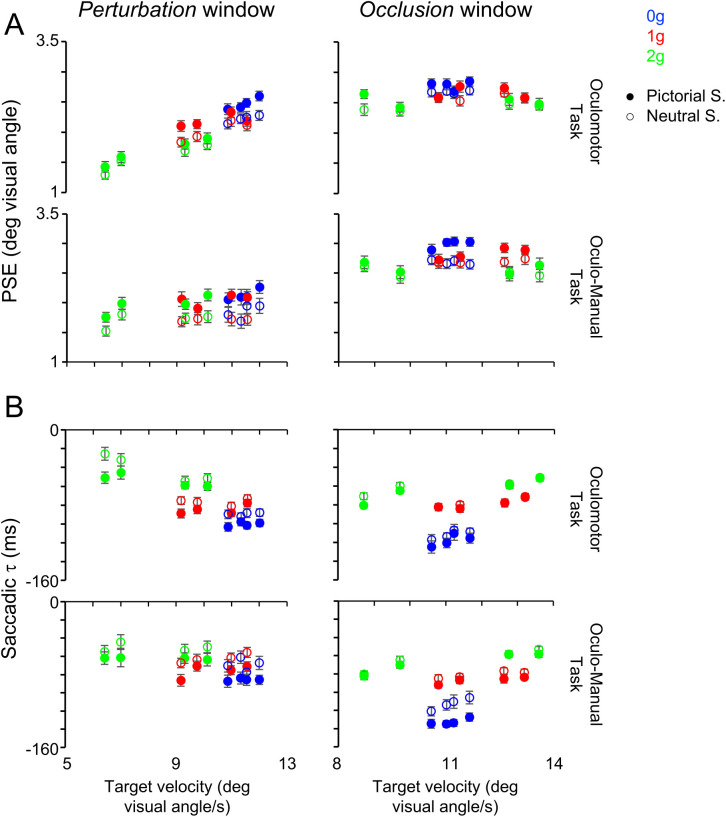
Relationships between saccadic indexes and target velocity. Each panel represents data of each oculomotor index with four subplots. The left column refers to the *perturbation* window and mean (± SEM) saccadic index values are plotted against the mean target velocity. The right column refers to the *occlusion* window and mean (± SEM) saccadic index values are plotted against the target velocity at the occlusion onset. The upper row represents data collected from subjects performing the Oculomotor Task while the lower row from subjects performing the Oculo-Manual Task. In each subplot, filled circles refer to data obtained with the Pictorial Scenario, open circles with Neutral Scenario. Blue, red and green symbols are related to 0g, 1g and 2g trials, respectively. **A)** PSE, post saccadic error, B) saccadic τ.

**Fig 7:**
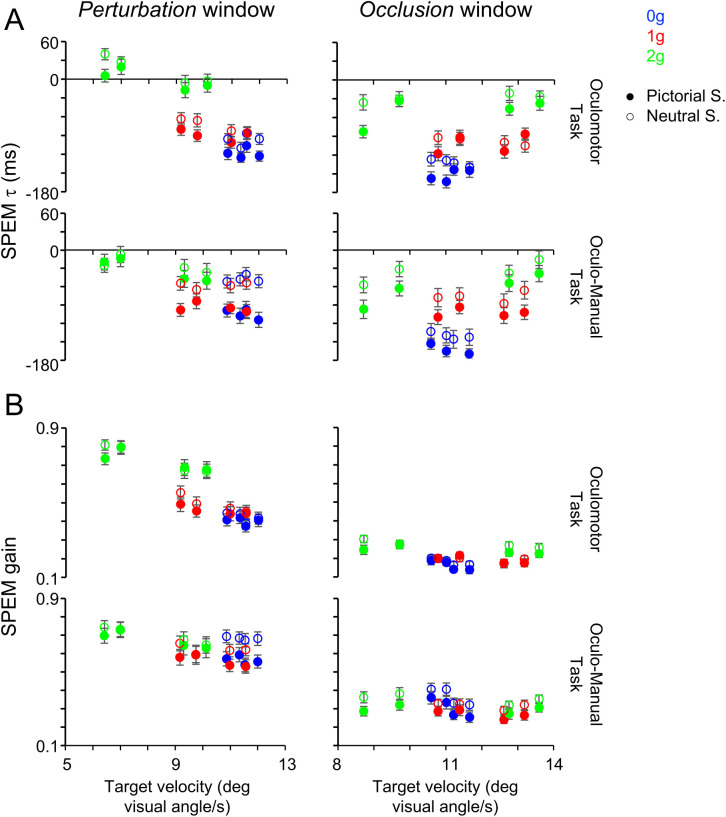
Relationships between SPEM indexes and target velocity. **A)** SPEM τ and **B)** SPEM gain. Same layout as Fig 6.

The ANOVA models revealed a strong dependency of all oculomotor indexes on Gravity Level and on several of its interactions, mainly with Window, Scenario and Task. Although these results, at face value, may be taken as evidence for strong effects of the manipulation of the gravity acceleration on the oculomotor behavior, our experimental design was partly confounded by the fact that acceleration levels covaried with the target velocities during the *perturbation* and *occlusion* windows (see Table 1). That is, the differences in the oculomotor performance across Gravity Levels could be as well accounted for by the changes in target velocity. To disentangle these effects, oculomotor indexes were directly compared between: 1) conditions with the same gravity level but different target velocity, and 2) conditions with similar target velocity but different gravity levels (Figs 6 and 7).

During the *perturbation* window, the percentage of significant differences (p < 0.010) between conditions with different velocity and same gravity level found for each oculomotor index, across combinations of Task and Scenario were 27.8% (10/36), 8.3% (3/36), 5.6% (2/36) and 22.2% (8/36) for post saccadic error, saccadic τ, SPEM τ and SPEM gain, respectively. Conversely, the percentage of significant differences between conditions with similar velocity and different gravity were 15.4% (8/52), 38.5% (20/52), 30.8% (16/52) and 21.2% (11/52) for post saccadic error, saccadic τ, SPEM τ and SPEM gain, respectively.

During the *occlusion* window, the proportions of significant differences between conditions with different velocity but the same gravity level, observed for each oculomotor index across Task and Scenario combinations, were 28.2% (11/39), 7.7% (3/39), 5.1% (2/39) and 20.5% (8/39) for post saccadic error, saccadic τ, SPEM τ and SPEM gain, respectively. In contrast, the percentage of significant differences observed between conditions characterized by similar velocity but different gravity level were 21.8% (12/55), 30.9% (17/55), 40.0% (22/55) and 27.3% (15/55) for post saccadic error, saccadic τ, SPEM τ and SPEM gain, respectively.

Further, we performed χ^2^ tests of independence for each oculomotor index to assess differences in the proportion of significant paired t-tests between equal gravity and different velocity and different gravity and similar velocity conditions. The analysis revealed a significant difference for saccadic τ and SPEM τ during both windows (all p ≤ 0.007), indicating that the fraction of significant tests was greater with different gravity levels compared to different velocity conditions. Similar trends, albeit not significant, were observed in the fractions of significant tests for SPEM gain during the *occlusion* window (p = 0.453). Instead, the opposite trends, whereby higher fractions of statistically significant comparisons occurred with different velocities and equal gravity conditions, were evident only with the post saccadic error during both *perturbation* and *occlusion* windows (p = 0.157 and p = 0.478, respectively) and with the SPEM gain during the *perturbation* window (p = 0.905), but they failed to reach statistical significance.

### Adaptation of oculomotor indexes to the experimental conditions

With respect to the possibility that subjects adapted their oculomotor performance while experiencing the experimental manipulations, analysis of the time courses of the mean oculomotor indexes among subjects for each experiment conditions indicated that learning effects were sporadic at best, being evident only for few experimental conditions and oculomotor indexes. Indeed, the double exponential fits to the time series of the oculomotor indexes never reached the statistical significance cut-off of p < 0.01 over 96 experimental conditions*oculomotor indexes, and we observed an R^2^ greater than 0.3 only in 10 cases.

### Interception performance

**Fig 8:**
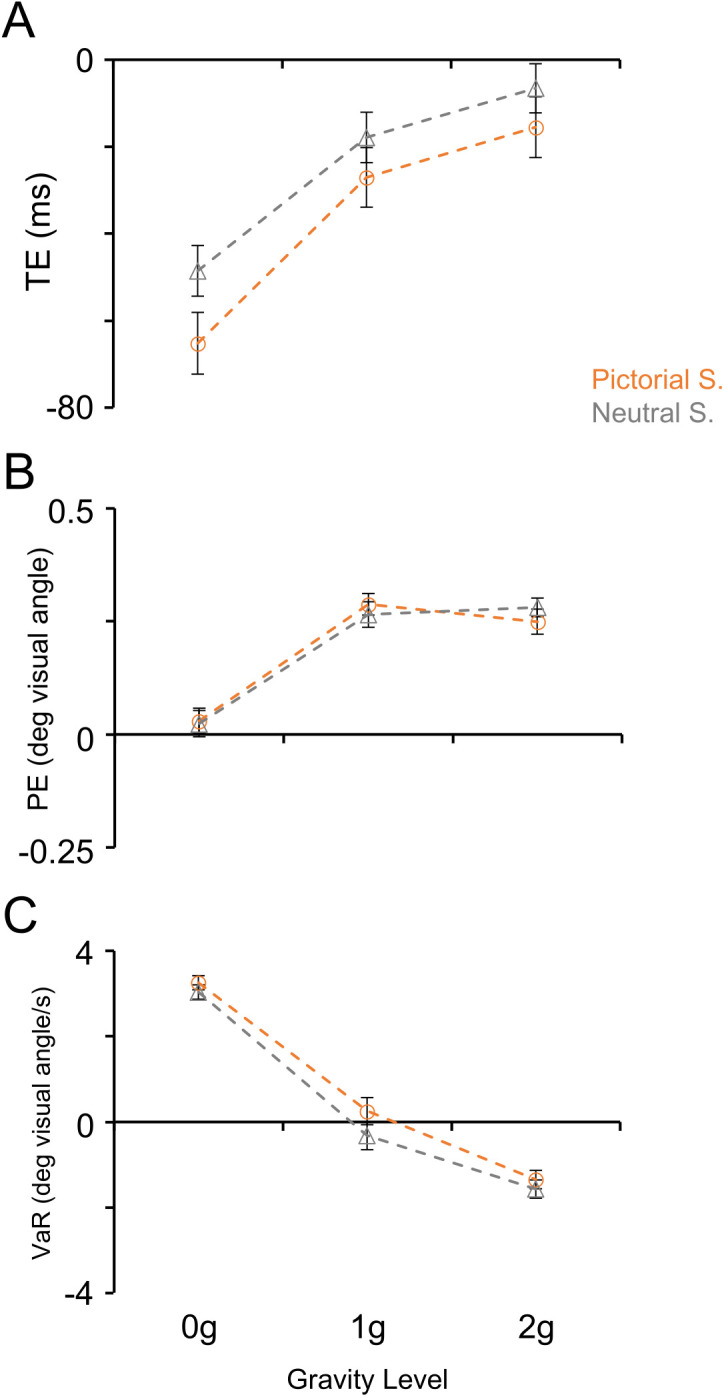
Subjects’ interception performance in the Oculo-Manual Task. The orange dashed lines and the open circles denote the interception parameters’ values (mean ± SEM) obtained with the Pictorial Scenario; the grey dashed lines and the open triangles those obtained with the Neutral Scenario. A) Timing error (TE). B) Positional error (PE). C) Velocity at Response (VaR). Same layout as Fig 4 and Fig 5.

We characterized subjects’ interceptive behavior by way of mixed ANOVAs on the Timing error, Positional error and Velocity at response (see Fig 8 and Table 5). A common finding emerging from these analyses was that Gravity Level represented the major factor accounting for the interceptive performance across experimental conditions, as indicated by the highly significant main effects reported for all three indexes in Table 5. In general, the changes in the interceptive performance related to the gravity level, seemed compatible with the use of a priori knowledge of gravity effects on the target motion. For example, the distribution of Timing error values across gravity levels denoted significantly stronger anticipation of the interceptive responses to 0g targets compared to accelerated 1g (paired t-test: t_(31)_ = 13.854, p < 0.001) and 2g (paired t-test: t_(31)_ = 17.149, p < 0.001) targets. In addition to the Gravity Level, Timing error values were significantly influenced by the type of Scenario, as participants produced earlier interceptive responses with the Pictorial Scenario compared to the Neutral Scenario, and by the three-way interaction Scenario*Gravity Level*Scenario Order (Table 5, Fig 8A).

**Table 5:** Results of repeated measures ANOVA on the indexes characterizing the interceptive performance (in bold p < 0.010, Greenhouse-Geisser corrected). Although the p-values reported are Greenhouse-Geisser corrected, to simplify the layout of the table and improve its readability, degrees of freedom (dof) are reported uncorrected, once for all the ANOVA tests. TE, Timing error; PE, Positional error; VaR, Velocity at response.

		TE			PE			VaR		
Factor	dof	F	p	η^2^	F	p	η^2^	F	p	η^2^
S	1,30	11.0	**0.002**	0.27	< 0.1	0.959	< 0.01	16.8	**< 0.001**	0.36
GL	2,60	219.2	**< 0.001**	0.88	64.0	**< 0.001**	0.68	184.7	**< 0.001**	0.86
SO	1,30	< 0.1	0.917	< 0.01	0.3	0.610	0.01	0.1	0.737	< 0.01
S*GL	2,60	3.3	0.061	0.10	1.8	0.184	0.06	6.5	**0.005**	0.18
S*SO	1,30	2.8	0.106	0.09	1.1	0.296	0.04	0.1	0.781	< 0.01
GL*SO	2,60	3.0	0.064	0.09	0.2	0.851	0.01	0.7	0.442	0.02
S*GL*SO	2,30	9.3	**0.001**	0.24	25.3	**< 0.001**	0.046	1.9	0.167	0.06

Positional differences across experimental conditions were much smaller than those observed for the Timing error (see Fig 8B). Nevertheless, we found a significant main effect of the Gravity Level, related to the slight overestimation of the responses to accelerated 1g (one sample t-test: t_(31)_ = 11.327, p < 0.001) and 2g (one sample t-test: t_(31)_ = 13.162, p < 0.001) targets, compared to the responses to 0g targets, which were not different from zero (one sample t-test: t_(31)_ = 1.261, p = 0.217). Alike the interceptive timing, Positional error values were influenced significantly also by the three-way interaction Scenario*Gravity Level*Scenario Order.

A monotonic relationship with the Gravity Level emerged clearly also with the values of the Velocity at response, accounting for the highly significant main effect of this factor (Table 5, Fig 8C). Velocity at response values were close to zero, on average, when subjects intercepted 1g targets (one-sample t-test: t_(31)_ = 0.108, p = 0.915), denoting that the cursor was firmly in place at the time of the response. Instead, with 0g and 2g targets, Velocity at response values were positive (one-sample t-test: t_(31)_ = 19.391, p < 0.001) and negative (one-sample t-test: t_(31)_ = 6.968, p < 0.001), respectively, indicating that subjects, at the time of the button press, were making corrections of the mouse cursor position in opposite directions for these two trajectories, compatible with expectation of gravity effects on the target motion.

Velocity at response values were also influenced by the type of Scenario, with slightly higher values when the task was performed with the Pictorial than with the Neutral Scenario (significant main effect of Scenario in Table 5). Finally, the statistically significant two-way interaction Scenario*Gravity Level, indicated that differences in Velocity at response values between the two scenarios were statistically significant during 1g trials (paired t-test: t_(31)_ = 4.163, p = 0.002), but not in 0g and 2g trials (paired t-tests: t_(31)_ = 2.939, p = 0.056; and t_(31)_ = 2.416, p = 0.196, respectively).

## Discussion

In this study, we sought to assess the extent to which ocular tracking could be influenced by the degree of naturalness of computer-generated visual scenes and by the concurrent execution of a manual interception action, reflecting the use of shared a priori gravity information.

### Effects of naturalness of the visual environment on ocular tracking

One type of manipulation of the naturalness of the visual environment consisted of sudden changes of the acceleration profile of the visual targets, which made their kinematics less compatible with the effects of natural gravity. The rationale for this manipulation stemmed from earlier evidence that a priori knowledge of gravity effects can be used effectively for the predictive control of manual interceptions as well as of eye movements (for a review, see [27]). Earlier studies did show a systematic dependence of the oculomotor performance on the acceleration imposed to the visual targets, similar to what reported for manual interceptions [23,25,26,56]. However, they did not test directly whether these oculomotor changes could be also related to the target speed imposed by the experimental manipulations [57–60]. Here, we found that ocular tracking was also strongly influenced by the target acceleration, regardless of the availability of visual information about target motion. Ocular tracking indexes showed, in fact, significant monotonic trends with respect to the target acceleration, denoting stronger anticipation of smooth pursuit and saccadic movements during 0g trajectories compared to accelerated 1g and 2g trajectories, as well as higher pursuit gains for accelerated trajectories. These monotonic trends seemed to reflect bona-fide effects of the target acceleration changes rather than of the target velocity. In fact, the analysis of the experimental conditions where changes in target velocity could be effectively dissociated from those of the gravity level indicated a higher fraction of statistically significant differences between conditions with different gravity levels and similar velocities than between conditions with different velocity and identical gravity level for the saccadic τ and the SPEM τ, that is, the oculomotor indexes reflecting, perhaps, more directly the predictive aspects of the ocular tracking. This result may imply that, within our experimental setting, the temporal aspects of the ocular tracking were more influenced by gravity level changes, compatible with expectation of gravity effects. Instead, for the oculomotor indexes related to the metrics of the ocular tracking, i.e., the SPEM gain and the post saccadic error, our analyses could not discriminate between effects of the target velocity per se and those of the gravity level, suggesting that these aspects may be equally dependent on feedback signals based on the target position and velocity and on predictive signals, which could also reflect gravity expectations.

At the same time we should note that, like our previous studies with similar experimental settings, the differences in the behavioral responses between 1g and 2g accelerated motion did not seem consistent across oculomotor indexes or the 2g even bettered 1g responses [23,39,41]. We could attribute this issue to the poor sensitivity of the visual system to retinal acceleration [76,77], and to the possibility that visual context information provided by the Pictorial Scenario could not be sufficient to scale adequately retinal acceleration to external world acceleration. However, we cannot completely rule out the possibility that oculomotor tracking scales monotonically with the magnitude of the target acceleration. In effect, studies in which the target acceleration has been manipulated using arbitrary values (i.e., not scaled to gravitational acceleration within a given quasi-realistic context) have reported systematic changes in scalar oculomotor parameters (e.g., SPEM gain or catch‑up saccades) that could be related to target acceleration and velocity increase [78,79]. However, the degree to which acceleration or velocity per se influence the oculomotor behavior may depend on the specific setting of the behavioral task. In fact, while there is evidence that target acceleration can be extracted and represented within the predictive drive to pursuit given sufficient exposure to the motion [78], other work indicates that observers often fail to integrate acceleration into predictive estimates of time‑to‑contact, relying instead on extrapolation of final target velocity [80].

Notwithstanding this potential confound, the consistency of the present results with earlier reports with respect to the effects of the manipulation of the naturalness of the visual context, taken together with the results of the analysis dissociating the effects of target velocity and gravity level, do suggest that subjects’ ocular tracking performance might reflect a priori expectations of gravity effects [39,41,81,82], at least for time-dependent oculomotor indexes, even though more conclusive evidence may be gathered by future studies dissociating experimentally the effects of the target acceleration per se from those of natural gravity.

In addition to the parametric changes of the target acceleration, we manipulated the realism of the visual background and found that visual context information influenced greatly ocular tracking, regardless of the availability of visual motion information. The changes in the oculomotor performance were consistent with those reported by a previous study from our laboratory employing a similar set of experimental conditions [39]. In the current study, post saccadic errors were larger and τ values more negative in the Pictorial compared to the Neutral scenario during both the *perturbation* and the *occlusion* windows. These results may be compatible with a greater level of anticipation –and, therefore, a higher degree of predictability– of the target’s motion when visual cues aid in interpreting its trajectory [83]. Noteworthy, this trend was evident also when the target motion was visually occluded, as information gathered while the target was visible, along with a priori knowledge, may have been used to maintain an anticipatory strategy in the absence of visual feedback about the target motion [84,85].

Smooth pursuit gain values were lower in the Pictorial than in the Neutral Scenario during both *perturbation* and *occlusion* windows. This result appears in line with previous findings that textured backgrounds can fragment eye tracking, by reducing the pursuit gain and increasing the number of saccades [86,87]. Moreover, in an earlier study by Delle Monache and colleagues (2019) [39], we reported an interaction effect, during the *perturbation* window, between the gravity level and the realism of the visual scene that was not replicated by the present study, where we observed a certain influence of gravity level in both scenarios, although to a slightly different extent. We may explain these differences between the earlier and the current study by assuming that participants were attempting to predict the target reappearance beyond the occluder, rather than tracking it, since the contour of the occluder cued where the target would reappear. In fact, in our previous study –where targets were not concealed behind a visual occluder but simply vanished for the same variable intervals as the present study– we found higher pursuit gains.

In other words, the occluder could have represented an attentional *attractor*, by providing cues about the onset and the spatial extent of the occlusion. By using these cues, participants may have shifted their gaze earlier to the occluder, rather than approaching it at the same speed as the target. Compatible with this view, Villavicencio and colleagues (2024) [88] observed higher pursuit gains when the occlusion onset was unpredictable compared to when it was predictable, likely because of a strong reliance on sensory information than on predictive mechanisms. Indeed, the findings reported by Villavicencio and colleagues (2024) [88] may help reconciling the differences we found between our previous study and the current one, since in the current study the onset of the occlusion was much more predictable than in the previous study. Thus, the predictability of the occlusion in the current experiment might explain the higher gains we observed during the *perturbation* window in the previous compared to the present study.

Another possibility to consider is that the occluder may have also worked as a *distractor*, independently of the type of visual scenario. It is known, in fact, that distractors can disrupt attention and influence saccadic movements [89–91], thereby potentially inducing saccadic errors. Smooth pursuit indexes (SPEM τ and SPEM gain values), however, were consistent with our previous study, showing stronger dependence on the target’s law of motion in the Pictorial than in the Neutral Scenario. The discrepancy that saccadic and smooth pursuit eye movements were affected differently by the interaction of the target kinematics with the type of visual scenario could be, in part, explained by the fact that, while distractors can also affect smooth pursuit [92], their influence is more pronounced during the initial open-loop phase. The influence of the distractor is, in fact, suppressed by selective attention mechanisms during the subsequent closed-loop phase driven by sensory signals, especially when, like in our study, the distractor’s position is rather predictable [93].

It must be pointed out, however, that, in the present experiment, the occluder initially fell in the peripheral vision, ruling out any distractor effect earlier on in the trial. As the target reached the *perturbation* window not less than 1.8 seconds later –i.e., long after the 450 ms open-loop phase subject to the influence of distractors [94]– its distance from the occluder was at least ~6.5 visual degrees, implying that the occluder image was still projected to the parafoveal region [95]. Thus, we may suggest that any potential distractor effects on the smooth pursuit movements were likely suppressed by the time the target reached the *perturbation* window, perhaps explaining why smooth pursuit indexes were not as affected by the introduction of the graphic occluder.

### Effects of the naturalness of the visual environment on the interceptive responses

With respect to the interceptive performance of the participants performing the Oculo-Manual Task, we found general agreement with previous findings based on similar manipulations of the visual target law of motion [23,96,97]. In fact, timing and spatial errors showed that 0g trajectories were underestimated compared to accelerated (1g and 2g) trajectories, a result considered compatible with gravitational expectation [23]. The distributions of mouse cursor velocities at the time of button presses were compatible with gravitational expectation as well, by denoting underestimation of 0g trajectories landing positions and overestimation of the 2g ones. Similar to Bosco et al. (2012) [23], we did observe values that were close to zero (i.e., no final corrections on the estimates of the targets landing position) for 1g trials, with evident rightward or leftward corrective movements during 0g and 2g trials, respectively.

### Influence of the concurrent interception task on ocular tracking

The execution of concurrent tasks may influence various aspects of oculomotor control, as clearly shown by the seminal findings by Yarbus [42]. Indeed, oculomotor control processes can be affected by the expected outcome of a specific behavioral strategy [98–100], by the nature of the concurrent task [101] and by its computational demands [102,103]. Moreover, there is evidence that performing two demanding tasks simultaneously may reduce the quality of ocular tracking [43,45–47]. Conversely, oculomotor strategies competing for attentional resources may introduce time gaps in cognitive tasks [104].

With respect to the concurrent execution of an interceptive task, it is known that ocular tracking performance can influence the interception accuracy [52,105], by way of a corollary discharge of the oculomotor commands, exemplifying a direct link between the precision of the ocular tracking and the performance of a specific concurrent task. This is particularly interesting from the perspective of the neural control mechanisms involved. For example, both ocular tracking and manual interceptive control involve predictive mechanisms, but the degree to which predictive information may be shared or weighted differentially between the two control systems is still a matter of debate.

In this regard, internal models of the physical properties of the environment appear to contribute critically to the predictive estimates of the timing of interceptive movements in humans [106–108], in non-human mammals [109,110], and even in some invertebrates [111]. Similar evidence that predictions about the object motion may be based on internal models of gravity effects has been gathered also for oculomotor control [39–41], suggesting that ocular tracking and manual interception share the same predictive information. However, it is not clear whether this predictive information is engaged with a similar temporal course between the two tasks.

A recent study from our group may give some indication about the time course with which internalized information about gravity may be engaged by the predictive processes underlying the control of interceptive timing [24]. In this study, we modelled the timing performance of subjects intercepting looming stimuli in a quasi-realistic 3D virtual reality setting, with Bayesian regression models which included a predictor related to an internal estimate of gravity effects, which could be engaged at successive time-points from the beginning of the visual motion up to 600 ms thereafter. The results of this analysis suggested that subjects relied exclusively on optical information during the first 450 ms of the descending trajectory (i.e., up to 350–650 ms before the nominal interception point, as target motion was comprised between 800 and 1100 ms), after which participants engaged and relied mostly on the gravity prior.

In our current study, the changes in oculomotor behavior might be used to infer the time-point at which the transition from sensory information to prior expectations occurred. Specifically, the significant three-way interaction effects of Window*Gravity Level*Task observed with all oculomotor indexes (Figs 4 and 5 and Tables 2 and 3), indicated differences between subjects performing either the Oculomotor or the Oculo-Manual Task with respect to the dependence of the oculomotor behavior on the gravity level during the *perturbation* compared to the *occlusion* windows. In fact, during the *perturbation* window, with visual feedback of the target motion, the dependence of saccadic and smooth pursuit parameters on expectation of gravity effects was clearly evident in subjects performing only ocular tracking, but it was weaker for participants performing also the target interception. Later in the trial, during the *occlusion* window, similar monotonic trends with respect to the gravity level emerged for post saccadic errors, saccadic τ and SPEM τ values regardless of the task, even though task-dependent differences persisted for smooth pursuit gain values. These findings imply that participants performing the Oculomotor Task began relying strongly on gravitational expectations at least 950–1200 ms before the target’s nominal landing. In contrast, when interception was also required (as for participants performing the Oculo-Manual Task), the transition from sensory information to internal model predictions occurred only after the target occlusion (i.e., not earlier than 400–650 ms before target’s nominal landing). That is, the introduction of the interception task seemed to postpone the recruitment of the internal model of gravity until predictive mechanisms were enforced by the visual occlusion of the moving target, as if subjects increased reliance on sensory signals as long as they were available to exploit every bit of incoming information [88,112]. Although the obvious differences in the experimental design and analysis make it difficult to compare directly the findings of the two studies, the current analysis of the oculomotor indexes between the two behavioral tasks does suggest a temporal course for the recruitment of the internal model of gravity like that implied by the earlier interception study. Thus, oculomotor and manual interception control may rely on a common mechanism that engages predictions of an internal gravitational model with a similar temporal profile when the two tasks are performed concurrently.

### Limitations of the study

One technical limitation of the study may concern the small dimensional changes in oculomotor indexes compared to the size of the visual target, considering also the spatial resolution of the EyeLink 1000 system we used for measuring eye movements. To address this potential issue, we adopted a rather restrictive significance cut-off of p < 0.01 on all statistical analyses, thereby mitigating the occurrence of false positives. Moreover, most of the effects of interest showed very high significance levels (p < 0.001) denoting that, however small, dimensional changes in the oculomotor indexes were extremely consistent.

## Conclusion

In sum, the current study provided further support to the idea that expectation of gravity effects on the motion of visual objects can influence significantly ocular tracking, much alike what consistently reported for interceptive movements. Remarkably, a dissociation emerged between the time related aspects of the ocular tracking (signified by the SPEM and saccadic τ) which reflected expectation of gravity effects, and those related to the pursuit metrics (SPEM gain and post saccadic error) which reflected the trajectory kinematics. By manipulating the realism of the visual context, we also substantiated earlier findings that pictorial cues informing about the environment naturalness can reinforce gravitational expectations, particularly in the absence of visual motion information. Finally, concurrent execution of the ocular tracking and of the interceptive task provided interesting insights on the temporal recruitment of the internalized gravity information, since gravitational expectations were applied consistently –even before predictive mechanisms were enforced by the target occlusion– by subjects performing only ocular tracking, whereas concurrent execution of the two tasks postponed the full recruitment of the internal gravity model to the period of visual occlusion, by increasing the reliance on visual information as long as it was available.

## Supporting information

S1 File Oculomotor indexes for *perturbation* (population_PERT.xlsx) and *occlusion* (population_OCCL.xlsx) windows and interception indexes (population_INTERCEPT.xlsx). Columns starting with PS and NS denote data from Pictorial and Neutral Scenario blocks, respectively. In the oculomotor indexes files, 0 and 1 values in the task column correspond to Oculomotor Task and Oculo-Manual Task, respectively. PSE, post saccadic error; SACCTAU, saccadic τ; SPEMTAU, SPEM τ; SPEMGAIN, SPEM gain.
(ZIP)
